# Sleep-related hypermotor epilepsy in a patient with mucopolysaccharidosis type III

**DOI:** 10.5935/1984-0063.20200113

**Published:** 2021

**Authors:** Anna A. Abramova, Hrayr P. Attarian, Snezhana M. Dolgova, Alexandra I. Belyakova-Bodina, Elena V. Iakovenko, Amayak G. Broutian

**Affiliations:** 1 Research Center of Neurology, Epilepsy Unit with Laboratory of Clinical Neurophysiology - Moscow - Russia.; 2 Northwestern University Feinberg School of Medicine, Center for Sleep Disorders - Chicago - Illinois - United States.; 3 Research Center of Neurology, Department of Neurogenetics - Moscow - Russia.

**Keywords:** Sleep-related Hypermotor Epilepsy, Mucopolysaccharidosis, Sanfilippo Syndrome, Hypermotor Seizures, Parasomnias

## Abstract

Both non-epileptic sleep disturbances and epilepsy are common in patients with mucopolysaccharidoses (MPS), so diagnosis of sleep-related hypermotor epilepsy in these patients is a tackling issue. We present a case of an adult patient with MPS IIIB (Sanfilippo syndrome), who presented with numerous nocturnal events of sudden awakening and hypermotor behavior, which had been previously regarded as parasomnias. Overnight video-EEG captured numerous stereotypical seizures with ictal pattern in the frontal regions, which led the diagnosis of SHE. The patient was started with carbamazepine, which resulted in a substantial reduction in the number of seizures. Our report provides further support for use of overnight video-EEG in the differential diagnosis of sleep-related disorders in MPS, yet true incidence of SHE in MPS patients remains unknown.

## INTRODUCTION

Various sleep disturbances are common in patients with mucopolysaccharidoses (MPS), including non-epileptic events such as parasomnias or sleep apnea. They are especially prevalent in MPS type III (Sanfilippo syndrome), which is characterized by predominant involvement of the central nervous system. Sleep-related hypermotor epilepsy (SHE), previously known as nocturnal frontal lobe epilepsy (NFLE), is an epileptic syndrome characterized by hypermotor seizures (HS) during sleep, causing sleep disruption in patients with MPS. Only a few cases of SHE have been described in patients with MPS, yet its true incidence remains unknown. We report another case of SHE in a patient with MPS IIIB, which provides further support for extended EEG monitoring during sleep in evaluating sleep complaints in MPS.

## CASE REPORT

An 18-year-old female presented to our clinic with a history of disrupted sleep caused by sudden brief awakenings with hypermotor activity, lasting for 5-10 seconds, followed by muscle soreness. These episodes first started at the age of 6, increasing from 5 to almost 60-70 per night by the age of 16. No events occurred during wakefulness.

Her mother first noticed poor coordination, disturbance of articulation and fine motor skills when the patient was three years old. Gait impairment and cognitive deterioration progressed over the course of the following years. At the time of admission, the patient was a college student, experiencing fatigue, attention decline, and affective lability that had a severe impact on her studies. Her family history was unremarkable.

Upon admission, clinical examination revealed dolichocephaly, narrow forehead, thick eyebrows with synophris, hypertrichosis, prominent philtrum, broad nasal tip, and a thickened lower lip. Neurological examination revealed signs of moderate dysarthria and coordination impairment. Abdominal ultrasound exam revealed hepatomegaly. Brain MRI showed diffuse cerebral atrophy. The level of urinary glycosaminoglicans was increased up to 14.3mg/ mmol (normal range 0-8.3mg/mmol). Two compound heterozygous mutations were revealed in the *NAGLU* gene through whole-exome sequencing (hg19). Both mutations were identified in the proband and confirmed by Sanger sequencing of the family trio (parentage verified). The first proband’s mutation was identified in exon 6 of the *NAGLU* gene (chr17:40695951C>T; c.1927C>T, NM_0000263.3). This variant was maternally inherited. Second heterozygous mutation was identified in exon 1 of the *NAGLU* gene (chr17:40688599G>A, c.309G>A, p.Trp103Ter). This variant was paternally inherited. Based on these findings a diagnosis of mucopolysaccharidosis (MPS) type IIIB was made.

**Figure 1:**
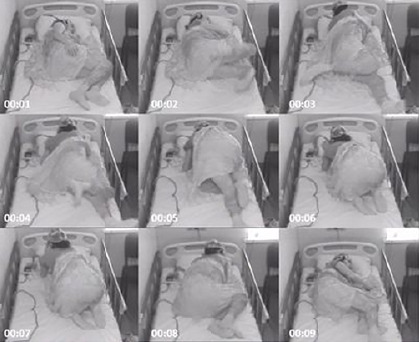
One of 21 focal stereotyped motor seizures captured during overnight video-EEG monitoring. The patient wakes up abruptly and presents with irregular hypermotor activity in all four limbs, which lasts for about 10 seconds.

**Figure 2:**
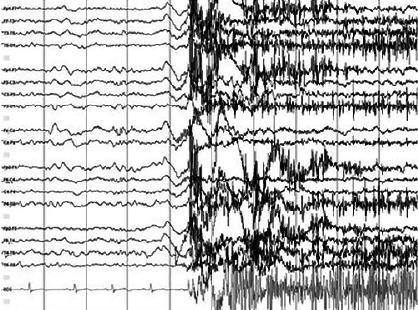
Overnight video-EEG monitoring: one of the seizures arising from non-REM sleep (stage 2). K-complex is followed by fast rhythmic activity in the frontal regions lasting for 0,3-0,5 seconds, before EEG is obscured with myographic artifacts.

Overnight video-EEG monitoring was performed, which captured 21 focal stereotyped motor seizures, characterized by sudden startled awakenings and irregular hypermotor activity in all four limbs (Figure 1). The events were preceded by rhythmic fast activity over the frontal regions (Figure 2). EEG monitoring also revealed interictal epileptiform activity in the frontal regions.

Based on seizure semiology and EEG abnormalities the patient was diagnosed with SHE. The patient was started with carbamazepine 800mg/day (400mg *b.i.d.*), leading to over 80% drop in seizure frequency. After one month, daily dose of carbamazepine was slowly increased to 1,200mg/day followed by a substantial reduction in the number of seizures to 1-2 per week.

## DISCUSSION

MPS type III (Sanfilippo syndrome) is the most common type of mucopolysaccharidoses (estimated prevalence 0.3-4.1 cases per 100,000 newborns). A metabolic lysosomal storage disorder, MPS III has an autosomal recessive inheritance. It consists of several subtypes depending on the site of the mutation and the following enzymatic deficiency in heparan sulfate catabolism. Our patient was diagnosed with MPS IIIB (OMIM #252920), associated with a mutation in the *NAGLU* gene, which encodes α-N-acetylglucosaminidase on chromosome 17q21^1^; c.1927C>T mutation is considered to cause an attenuated clinical phenotype of the disease^2^. Accumulation of heparan sulfate accounts for predominant nervous system involvement in MPS type III, while in other MPS types impaired hydrolysis of chondroitin/dermatan sulfate leads to a more pronounced somatic presentation of the disease^3^.

Pathogenesis of neurological manifestations in MPS III involve both neuronal and non-neuronal cells in the central nervous system (CNS)^4^. Accumulation of heparan sulfate contributes to lysosomal dysfunction, autophagy impairment, disruption of mitochondrial functioning, and oxidative stress^3^. Thus, MPS has a microglial neuroinflammation component, which has been shown to have common molecular pathways with epileptogenesis^6^.

MPS III is characterized by a three-stage course^7^. During the first stage (1-3 years of age), elements of cognitive decline (speech or motor development delay) emerge, which become more prominent during stage two (typically lasts for 7-10 years). These are accompanied by severe behavioral and sleep disturbances, including elements of autistic-like, aggressive outbursts. Third stage is characterized by severe dementia and further neurological, and somatic decline. Seizures may occur during the second and third stages of the disease^8^.

Since behavioral and sleep problems are burdensome for both patients and their parents, they can be the only complaints in attenuated MPS IIIB. Patients of this group are often misdiagnosed with autism spectrum disorders or idiopathic developmental delay, which was delayed proper diagnosis in our patient^8^.

Seizures in MPS patients are mostly tonic-clonic, although myoclonic, absence, focal seizures, non-convulsive status epilepticus, and nocturnal myoclonic jerks have been reported. Seizures, to some extent, are markers for rapidly progressing disease^9^.

In a cohort study by Delgadillo et al. (2013)^10^ including 55 MPS III patients, 45% of all patients experienced seizures, with the mean age of seizure onset at 8.7 (2.5-37) years. Patients with MPS IIIB tended to have a later seizure onset than other MPS IIIs, with mean age of seizure onset around (5.5-37) years. Most reports are of tonic-clonic seizures that responded well to monotherapy.

In a large metanalysis by Scarpa et al. (2017)^9^, the highest prevalence of seizures was observed in patients with MPS III (26-52%). As expected, incidence of seizures increased along with neurocognitive deterioration. In patients with MPS the severity of sleep disturbances varies greatly with an approximate prevalence rate of 87-92%^11^. It is usually characterized by deterioration of circadian sleep wake cycles. Some children with MPS III tend to have longer sleep onset latencies and increased daytime drowsiness in comparison to healthy controls. In polysomnographic studies, MPS IIIA patients demonstrate shorter overnight sleep, decreased REM, and slow wave sleep^12^. Sleep-related seizures could cause sleep impairment, which in turn can result in daytime somnolence, hyperactive behavior, and shortened attention span, and other severe behavioral disorders^9^.

Actigraphic recordings in children with MPS III demonstrated multiple awakenings (87.5%), with most of the children exhibiting disruptive behavior, such as screaming, laughing, singing or even running out of their bedroom^13^. Nevertheless, SHE should be on the differential diagnosis of sleep-related events in this population. In our case, the patient was diagnosed with SHE on the basis of the following features:

1. Episodes with abrupt awakening from sleep with asymmetric hyperkinetic movements;
2. Brief duration and stereotypical semiology;
3. Ictal and interictal epileptiform activity in the frontal regions.

Occurrence of SHE with typical hypermotor seizures in MPS has been described before. Bonanni et al. (2012)^14^ presented a case of frontal non-convulsive status epilepticus in a 7-year-old-female with MPS II, who developed severe cognitive impairment, which was regarded as part of the natural course of the disease. EEG was performed many months later, revealing continuous rhythmic spike-wave activity in the frontal regions. Therapy with ethosuximide substantially decreased the ictal activity and resulted in marked clinical improvement. At the age of 10 symptoms reappeared. Long-term video EEG monitoring demonstrated episodes of abrupt awakening, hyperkinetic automatisms, vocalization, and hyperventilation (an overall of 269 episodes per night). Therapy with clobazam and carbamazepine controlled the seizures completely^15^.

Another case of SHE in an 11-year-old patient with MPS IIIA, was also reported by Bonanni et al. (2014)^15^. Long-term video EEG captured 74 episodes of sudden awakening and hyperkinetic automatisms. Again, administration of clobazam led to a significant reduction in the frequency of seizures.

Differential diagnosis of paroxysmal motor events in sleep may be challenging. Unusual semiology of seizures in SHE as well as lack of any specific ictal EEG patterns (or them being obscured by movement and electromyographic artifacts) result in SHE being mistaken for psychogenic non-epileptic seizures or parasomnias^17^.

Although epileptic seizures are known to be frequent in MPS, SHE is rarely documented as a leading cause of sleep disturbances in these patients. We suggest using long-term video EEG monitoring instead or in combination with polysomnography in order to identify the etiology of paroxysmal events at nighttime. Timely diagnosis of epilepsy leads to substantial alleviation of neurological and behavioral symptoms, following sleep normalization.

## CONCLUSION

We suggest that in all cases of paroxysmal episodes of sleep disturbance in patients with MPS III video-EEG monitoring should be performed in order to rule out epilepsy. It should be recognized that frequent seizures, if not managed with AEDs, could result in worsening of neuropsychological status. Finally, we believe that association between sleep-related seizures and other non- epileptic sleep disorders in these patients deserves consideration.
